# Understanding Transient Ionic Diode Currents and Impedance Responses for Aquivion-Coated Microholes

**DOI:** 10.1021/acsami.3c08543

**Published:** 2023-08-11

**Authors:** Evaldo Batista Carneiro-Neto, Zhongkai Li, Ernesto Pereira, Klaus Mathwig, Philip J. Fletcher, Frank Marken

**Affiliations:** † Department of Chemistry, University of Bath, Claverton Down, Bath BA2 7AY, United Kingdom; ‡ Department of Chemistry, Federal University of São Carlos, Rod. Washington Luiz, Km 235, CEP, São Carlos 13565-905, São Paulo, Brazil; § imec within OnePlanet Research Center, Bronland 10, 6708 WH Wageningen, The Netherlands; ∥ University of Bath, Materials & Chemical Characterisation Facility MC^2^, Bath BA2 7AY, United Kingdom

**Keywords:** ionic diode, desalination, diffusion−migration, accumulation−depletion, electrochemical impedance spectroscopy

## Abstract

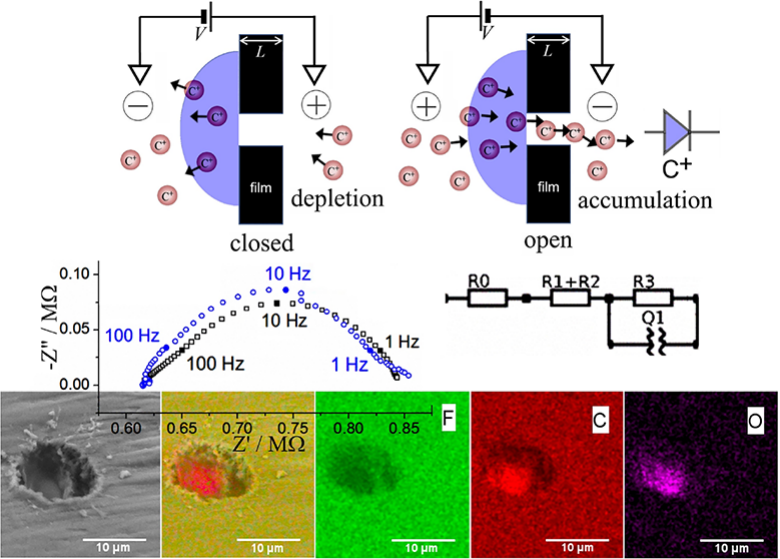

Ionic diode based devices or circuits can be applied, for example, in electroosmotic pumps or in desalination processes. Aquivion ionomer coated asymmetrically over a Teflon film (5 μm thickness) with a laser-drilled microhole (approximately 10 μm diameter) gives a cationic diode with a rectification ratio of typically 10–20 (measured in 0.01 M NaCl with ±0.3 V applied bias). Steady state voltammetry, chronoamperometry, and electrochemical impedance spectroscopy data are employed to characterize the ionic diode performance parameters. Next, a COMSOL 6.0 finite element model is employed to quantitatively assess/compare transient phenomena and to extract mechanistic information by comparison with experimental data. The experimental diode time constant and diode switching process associated with a distorted semicircle (with a typical diode switching frequency of 10 Hz) in the Nyquist plot are reproduced by computer simulation and rationalized in terms of microhole diffusion–migration times. Fundamental understanding and modeling of the ionic diode switching process can be exploited in the rational/optimized design of new improved devices.

## 1 Introduction

Ionic diodes are asymmetrically ion conductive devices based on nanopores,^1,2^ nanocones,^3−6^ micro/nano-fluidic systems,^7^ or based on microhole devices.^8^ By combining or coupling ionic diodes into ionic circuits,^9^ alternating current electricity can be employed to drive processes such as electroosmosis^10^ or water desalination avoiding side reactions at capacitive driver electrodes.^11^ Characteristic properties for ionic diodes are linked to the rectification ratio (the ratio of currents in “open” and “closed” states) and the time constant for switching of the diode state. The rectification ratio (as a function of applied voltage) is obtained experimentally, for example, by voltammetry under close to steady state conditions. In contrast, the diode switching time constant is a transient property accessible, for example, with chronoamperometry or with electrochemical impedance spectroscopy. For ionic diodes based on a Nafion ionomer film on a 10 μm diameter microhole, diode switching frequencies of typically *f*_diode_ = 10 Hz were observed from the “summit frequency” of depressed semicircular features in the Nyquist representation.^12^ This can be translated into a corresponding diode time constant with τ_diode_ = 1/ω_diode_ = 1/(2π*f*_diode_) = 16 ms in this case. Although electrochemical impedance spectroscopy studies have been previously reported for microhole ionic diodes, there has been no full analysis of the impedance data in terms of physical parameters, such as concentration profile changes linking to diode time constant and diode frequency. It is reported here that the finite element simulation approach allows the diode time constant and frequency parameters to be better understood.

**Figure 1:**
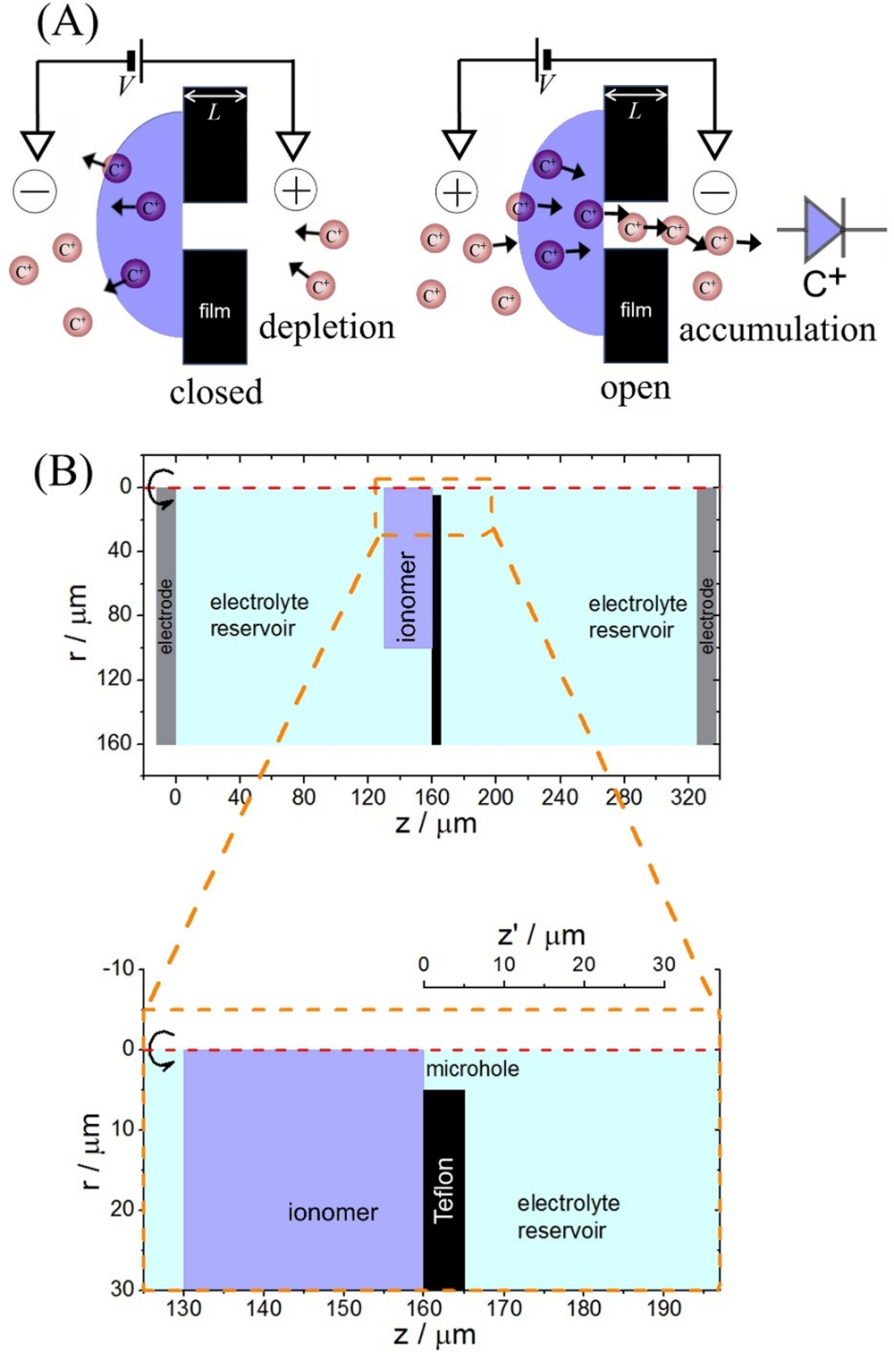
Representation of (A) the diode switching process and (B) the geometry employed in multiphysics simulations. Due to the symmetry around the axial axis (red dashed line) of the system, only a 2D model was considered (shown here as cross section of half of the cell = the asymmetric unit). The region contained inside the orange dashed rectangle is enlarged below to show the details of the microhole. The internal ionomer|water interface is defined as the starting point of the axis *z*′ (used later to represent the profile of the sodium concentration).

In this report, the ionomer Aquivion from Solvay (commonly applied in fuel cells and electrolyzers)^13,14^ has been selected for the assembly of ionic diodes on a 5 μm thickness Teflon substrate with a laser-drilled microhole (approximately 10 μm diameter). Aquivion offers good conductivity^15^ and opportunities for additive device manufacturing.^16^ Previously, films of ion conducting materials such as cellulose,^17^ M13 phage,^18^ nanocarbons,^19^ graphene oxide,^20^ or polyacrylonitrile^21^ have been employed in ionic diodes. The charge density of Aquivion (designed for fuel cell^22^ and energy applications^23,24^) is relatively high (approximately [−e^–^] = 1.16 mol dm^–3^ or −112000 C dm^–3^) to provide high cation conductivity while retaining processability and chemical stability. Figure 1A shows the operational principle based on a highly cation-conductive ionomer film asymmetrically deposited over the microhole in a Teflon substrate. Depending on the applied bias potential, either accumulation or depletion of aqueous electrolyte within the microhole region occurs, leading to the observation of “open” and “closed” diode states, respectively.

Multiphysics finite element computational models provide powerful tools for the simulation of electrochemical processes and data.^25^ From a modelist perspective, the ionic diode devices can be described by the simultaneous solution of the Nernst–Planck–Poisson equations with appropriate boundary conditions. This approach has been employed previously for example to investigate the ion current rectification in nanopores^26^ or the ion transport through charged conical micropores^27^ and for the ion transport in asymmetric ionomer film deposits over microholes.^28^ For the latter case, previous studies have been limited to steady state current data. Here, the transient current components and electrochemical impedance spectroscopy data are investigated. It is shown that the computer simulation model can reproduce the experimental data without the need to invoke nonphysical equivalent circuit models (these are used here only for comparison of data sets).

In this report, the processes in a cationic diode based on Aquivion coated 30 μm thick over an approximately 10 μm diameter microhole in 5 μm thick Teflon are investigated. Both steady state and transient currents (voltammetry, chronoamperometry, and electrochemical impedance spectroscopy) are investigated. Comparison of the finite element model with experimental data suggests that diffusion–migration processes in the microhole region determine the diode time constant.

## 2 Experimental Section

### 2.1 Chemical Reagents

Aquivion D98–25BS (25% w/w in water; equiv wt 980 g/mol SO_3_H; density 1.14 g cm^–3^; approximately charge concentration 1.16 mol dm^–3^) was purchased from Sigma-Aldrich. Absolute ethanol (99.97%) was purchased from VWR Chemicals. Sodium chloride (99.5%) was purchased from Fisher Scientific Ltd. Agarose powder was purchased from Melford Ltd. All chemicals were utilized as received without further purification. Aqueous solutions were prepared using ultrapure water from a Thermo Scientific water purification system, with a resistivity not less than 18.2 MΩ cm (20 ± 2 °C). The pH of 0.01 M NaCl was 6.9 ± 0.1.

### 2.2 Instrumentation

**Figure 2:**
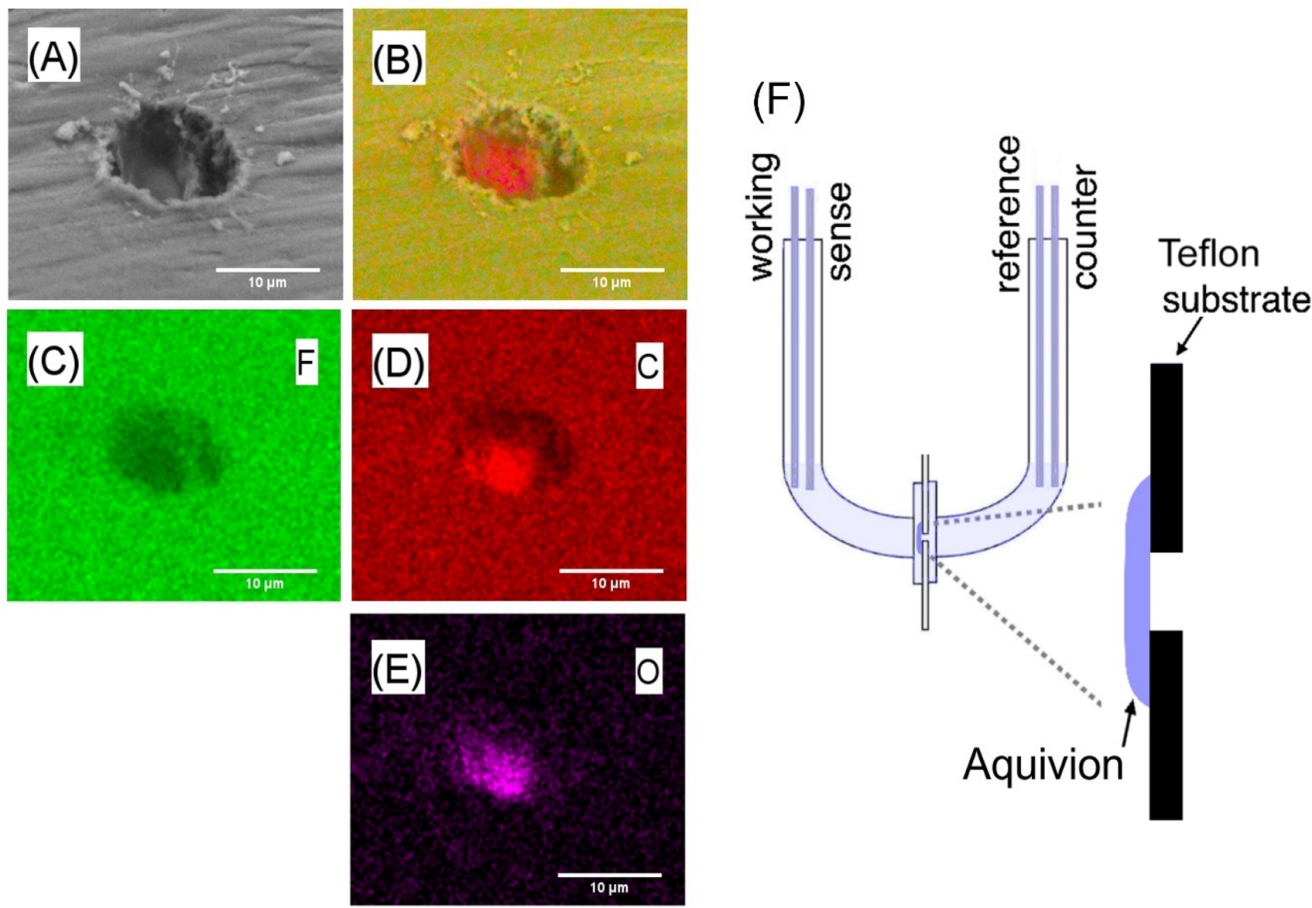
(A) Scanning electron microscopy (SEM, secondary electron) images of the backside of Aquivion-coated Teflon film with 10 μm microhole. (B–E) EDX mapping for F (mainly in Teflon) and C and O (mainly in Aquivion). Image (B) is layered EDX images combing (C–E). (F) Schematic of the experimental 4-electrode cell for voltammetry and for electrochemical impedance spectroscopy experiments. The ionomer is on the working electrode side.

Electrochemical measurements were performed with a 4-electrode configuration on a computer-controlled Ivium Technologies CompactStat potentiostat for cyclic voltammetry, chronoamperometry, and for electrochemical impedance spectroscopy. The electrochemical cell contained two cylindrical half-cells, which were separated by a Teflon thin film (5 μm thick) with a laser-drilled microhole of 10 μm diameter (see Figure 2; Laser Micromachining Ltd., St. Asaph, LL17 0JG, UK). The microhole region was coated asymmetrically with an Aquivion membrane. Briefly, the Teflon film was placed onto 1% agarose gel to allow Aquivion solution (20% v/v dilution of Aquivion solution in ethanol) to be applied from one side only. Once the ionomer film was dry, the modified Teflon film was peeled off the gel and used in the electrochemical measurements. Figure 2 shows typical scanning electron microscopy images with EDX elemental mapping for the Teflon film with a microhole and Aquivion film underneath. Image 2C corresponds to the fluorine elemental distribution with Teflon dominating. Image 2D shows the carbon elemental map with a stronger signal for Aquivion compared to that for Teflon. In contrast, Figure 2E shows that oxygen is more abundant in Aquivion. Figure 2F shows the experimental configurations for electrochemistry. Carbon rods of 1 mm diameter served as both working and counter electrodes, and silver wires (0.5 mm diameter) were applied as quasi-sense and quasi-reference electrodes. The symmetry of the system (with electrolyte solutions) ensured that the silver quasi-reference electrodes are always at equal potential. Silver quasi-reference electrodes help avoiding artifacts in impedance data due to frits in commercial reference electrodes. During all measurements, the working and sense electrodes were placed on the membrane side of the cell. Scanning electron microscopy (SEM) and energy-dispersive X-ray spectroscopy (EDX) were performed on a Hitachi SU390 variable pressure SEM with attached Oxford Instruments Ultim Max 170 mm^2^ EDX detector for the microhole imaging and on a Jeol JSM-7900F field emission SEM with attached Oxford Instruments Ultim Extreme 100 mm^2^ windowless EDX analyzer for the cross-sectional imaging.

### 2.3 Procedures

#### 2.3.1 Membrane Preparation

**Figure 3:**
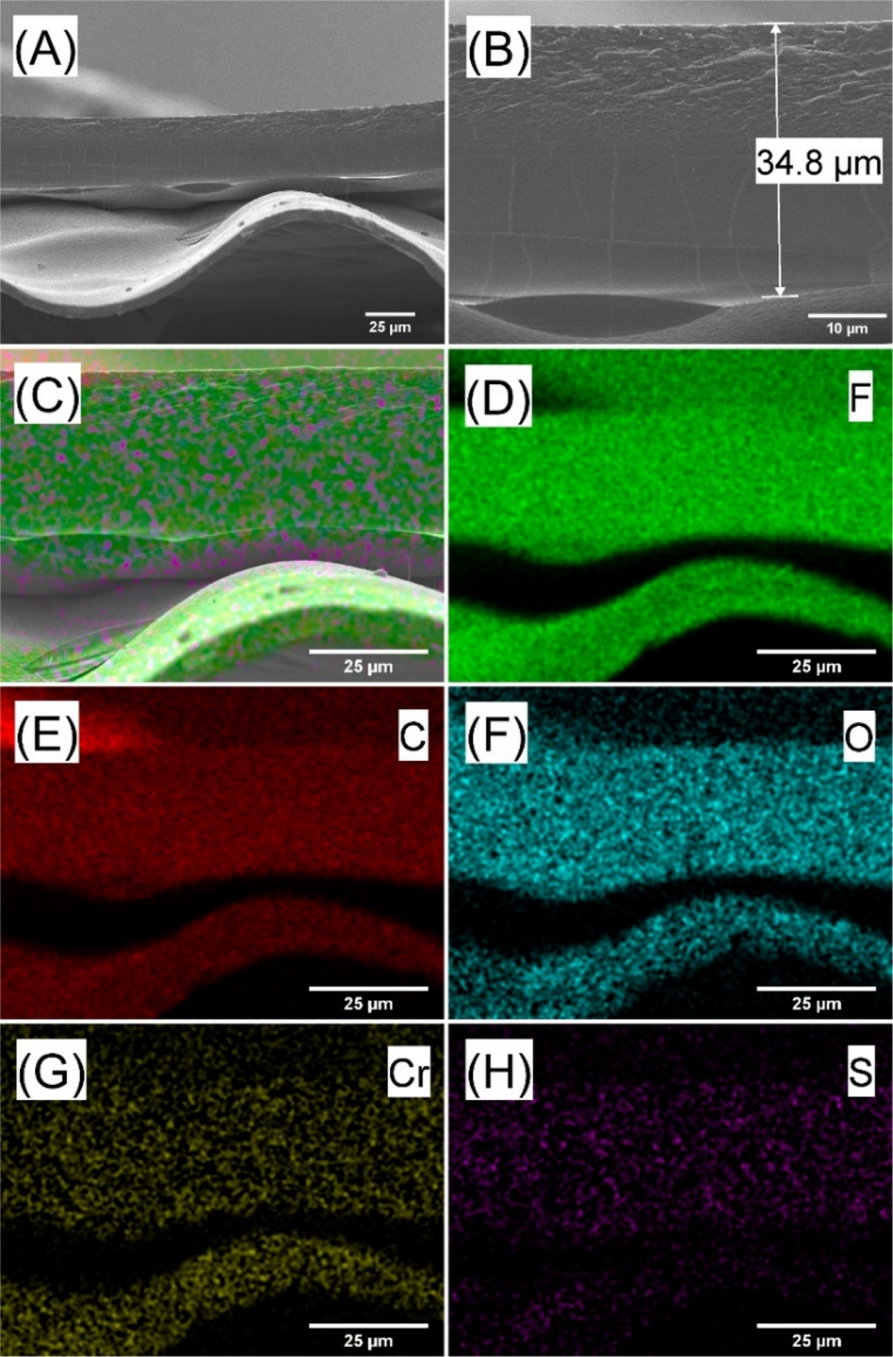
(A, B) Cross-sectional scanning electron microscopy (SEM, secondary electron, 5 nm chromium applied to suppress charging) images of a 5 μm Teflon film (approximately 5 μm thick) coated with Aquivion membrane (approximately 30 μm thick). (C–H) EDX mapping for F, C, O, Cr, and S. F is present in both materials, but S and O are mainly located in Aquivion. Image (C) is layered EDX images combing (D–H).

A 20% v/v dilution of Aquivion solution in ethanol was prepared to create a similar ionomer concentration to the previously reported studies on Nafion diodes.^12^ The Teflon film was placed on an agarose gel substrate to prevent the ionomer solution from penetrating through the microhole. A volume of 10 μL of the solution was then deposited onto the microhole region of the Teflon film to form a membrane by drop-casting. For SEM and EDX analysis, Aquivion membranes prepared with this methodology were coated with 5 nm of chromium metal to improve electrical conductivity and remove charging. Cross-sectional SEM and EDX indicated an Aquivion membrane thickness of typically 30 ± 5 μm (Figure 3).

#### 2.3.2 Electrochemical Cell Assembly

The electrochemical characterization of the Aquivion diode was performed in a “U-cell” with 4-electrode configuration (Figure 2F). A solution of aqueous 10 mM NaCl was placed into both reservoirs as electrolyte for performing cyclic voltammetry, chronoamperometry, and electrochemical impedance spectroscopy. The presence of the Aquivion membrane coated asymmetrically on the microhole in Teflon is shown in Figure 2.

## 3 Finite Element Simulation

In the computer simulation (COMSOL Multiphysics 6.0), the two-dimensional axisymmetric ionic diode geometry (Figure 1B) is formed by two identical cylindrical reservoirs connected by a single microhole (due to axial symmetry, the simulation is possible in 2D using the asymmetric unit in Figure 1). Each reservoir has a radius of 160 μm and a height of 160 μm. The microhole has a length of 5 μm and a radius of 5 μm and is covered on one side with 30 μm thickness ionomer film which extends 100 μm radially. The reservoir and microhole spaces were filled with a 10 mM NaCl solution (see parameters in Table 1). The ionomer film has a uniform fixed negative charge with an assumed concentration of 0.05 mol dm^–3^. This is lower compared to the Aquivion charge density of approximately 1.16 mol dm^–3^ and a compromise to achieve convergence and to avoid a much finer meshing and substantial additional computational time requirements.

**Table 1:** Summary of Parameters and Associated Values Defined in the Simulations

symbol	value	description
*D*_*Na*,*e*_	1.3 × 10^–9^ m^2^ s^–1^^a^	diffusion coefficient of Na^+^ in the electrolyte
*D*_*Cl*,*e*_	2.0 × 10^–9^ m^2^ s^–1^^a^	diffusion coefficient of Cl^–^ in the electrolyte
*D*_*Na*,*mem*_	6.5 × 10^–11^ m^2^ s^–1^^b^	diffusion coefficient of Na^+^ in the membrane
*D*_*Cl*,*mem*_	8.4 × 10^–10^ m^2^ s^–1^^b^	diffusion coefficient of Cl^–^ in the membrane
*c*_*mem*_	0.05 mol dm^–3^^c^	concentration of fixed negative charge inside the membrane
*z*_*i*,*k*_	±1^c^	ionic charge for Na^+^ and Cl^–^

a From ref (29).

b Parameters that are free; estimated in the impedance fitting process.

c Parameters that are fixed in the simulations.

Electroneutrality is maintained in the electrolyte
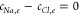
1and inside the ionomer
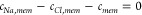
2

Here, *c*_*Na*,*e*_ and *c*_*Cl*,*e*_ are the sodium cation and chloride anion concentrations in the electrolyte. The parameters *c*_*Na*,*mem*_ and *c*_*Cl*,*mem*_ denote the sodium cation and the chloride anion concentrations in the ionomer. The parameter *c*_*mem*_ describes the concentration of fixed negative charges (assumed to be anionic sulfonate groups) inside the ionomer. The electrolyte potential is described by the Laplace equation.
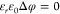
3

Here, *ε*_*r*_ is the relative dielectric constant, which in this case is assumed to be 80, ε_0_ is the vacuum permittivity constant, and φ is the local electrolyte potential. The distribution of concentrations inside the system is described by the Nernst–Planck equation.
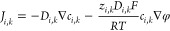
4
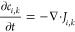
5Here, *c*_*i*,*k*_, *D*_*i*,*k*_, and *z*_*i*,*k*_ are, in this order, the flux density, the concentration, the diffusion coefficient, and the ionic charge of species *i* (e.g., Na^+^ or Cl^–^) in domain *k* (e.g., electrolyte or ionomer layer). *F* and *R* have their usual meanings, and *T* is the absolute temperature which in this case is 298.15 K.

At both ends of the domain (e.g. at *z* = 0 μm and at *z* = 325 μm for all *r*) it was assumed that both ionic concentrations were equal to the bulk concentration (i.e., 10 mM). A potential difference was applied between these external interfaces. The boundaries between the electrolyte and the ionomer film were defined with the Donnan potential condition, which considers the electrochemical potential to be the same on each side of the interface.
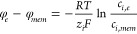
6

Here, the subindexes *e* and *mem* refer to the electrolyte or membrane, respectively. All the other boundaries were defined as null concentration flux and with zero surface charge. In all simulations, a first step was performed which consisted of solving the set of equations under steady-state condition and in the absence of applied potential. With this done, it was possible to calculate the equilibrium concentrations of sodium cations and chloride anions and the electric potential profile throughout the device with applied bias. Table 1 gives the important parameters with a brief description.

The current through the diode device is calculated by the integration of the normal ionic flux over one of the ends of the domain. At the boundary *z* = 325 μm and for 0 ≤ *r* ≤ 160 μm, the expression below was employed to give the net current.
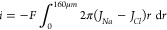
7

The equations are solved by employing the Finite Element Method (FEM, COMSOL Multiphysics 6.0). A mesh was built according to a predefined method with a maximum element size of 12 μm. At the interface between the electrolyte and the ionomer, the maximum element size was 0.10 μm, except for the interface inside the microhole, where the maximum element size was 0.01 μm, and at the rim of the microhole, where the maximum element size was 10^–4^ μm. This was necessary to ensure accurate calculation of the gradients in every region. The total mesh in the model was constituted by 119508 elements. The computational time spent in each simulation was about 12 h (on a personal computer with a processor Intel Core I7–8700 with 6 cores working at 3.20 GHz and 32 GB of RAM memory).

## 4 Results and Discussion

### 4.1 Aquivion-Coated Microhole Diodes: Experimental Data

**Figure 4:**
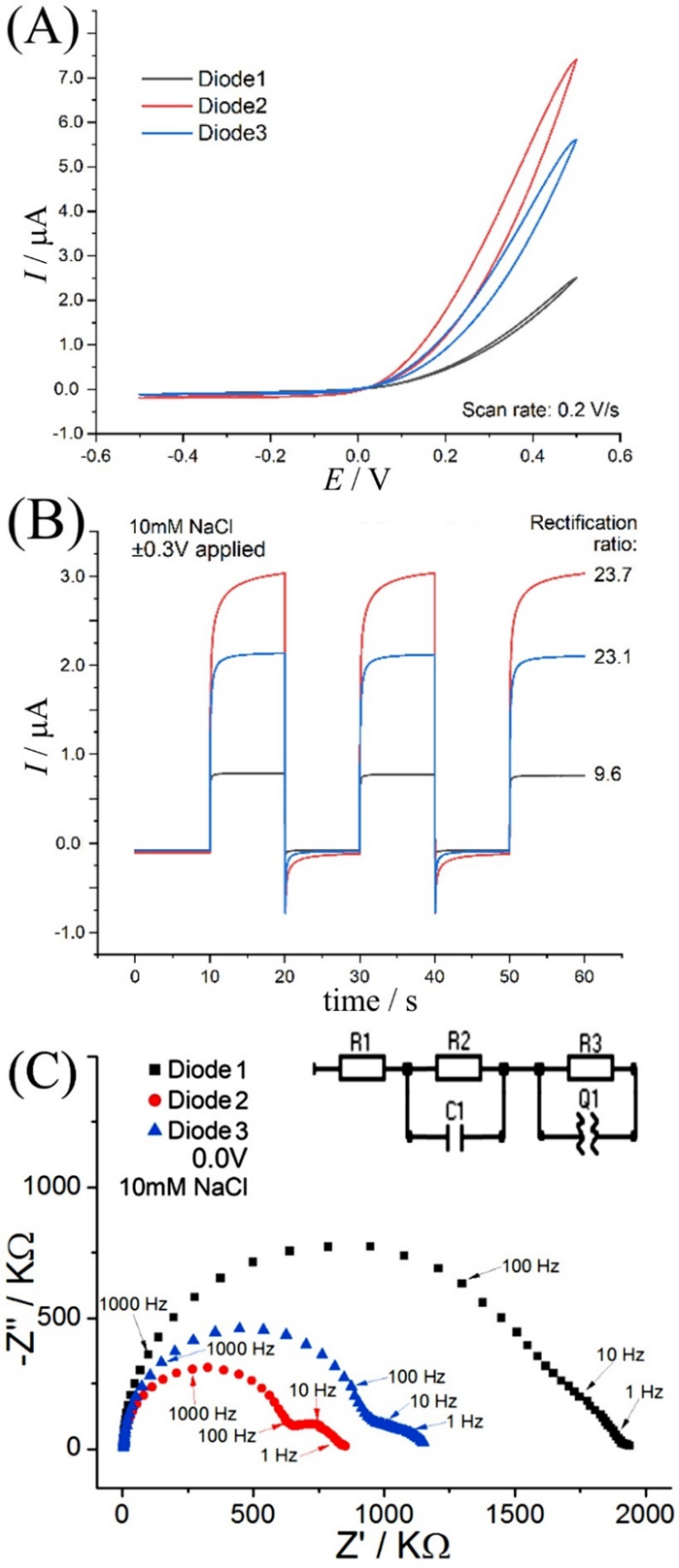
(A) Cyclic voltammograms (scan rate 0.2 V s^–1^) for three Aquivion microhole diodes in 0.01 M NaCl aqueous solution. (B) Chronoamperometry transient currents for the diodes with applied pulses of ±0.3 V. (C) Electrochemical impedance data for the diodes with an amplitude of 25 mV, bias of 0.0 V, and frequency range of 1 Hz to 100 kHz.

Typical cyclic voltammetry data for a 30 μm thick Aquivion film on a 5 μm Teflon film with a 10 μm diameter microhole are shown in Figure 4A for the 10 mM NaCl electrolyte. With a potential scan rate of 0.2 V s^–1^, data are close to steady state, although a current loop is observed in the positive potential range. In the negative potential range, the ionic diode is closed and currents remain low. In the positive potential range, accumulation of the electrolyte in the microhole causes lower resistivity and higher currents (the diode is open). Three typical current traces for three repeat ionic diode devices are shown. The variation in the observed currents can be attributed to (i) variability in the laser drilling and shape of the microhole (see Figure 2) and (ii) variability in the ionomer coating in terms of thickness and in terms of some penetration of ionomer into the microhole. Rectification ratio data were obtained by chronoamperometry at ±0.3 V (Figure 4B). Typical values range from 10 to 23. Perhaps interestingly, devices with the lower rectification ratio appear to be linked also to a faster switching time constant (*vide infra*).

The analysis of the diode behavior based on electrochemical impedance spectroscopy provides further quantitative detail. When performed in the closed state (negative potential range) or in the open state (positive potential range), the diode is essentially locked into low or high resistivity behavior (not shown). However, with a bias potential of 0.0 V, the diode switching process from open to closed can be further investigated. Data in Figure 4C show typical Nyquist plots with all three devices showing a first (high frequency) and a second (low frequency) semicircular feature. The higher frequency semicircle is associated with charging of the Teflon film. The lower frequency semicircle is associated with the diode switching.

The impedance data can be fitted and represented (in first approximation, see Table 2) by an electric circuit model (Figure 4) based on a serial resistor (*R*1), a capacitor describing the charging of the Teflon film (*C*1), a resistor describing ion current flow through the ionomer and microhole region at high frequency and without concentration polarization (*R*2), and a resistor (*R*3) constant phase element (*Q*1, *N*) representing the process of diode open/close switching. The electric circuit model fit is satisfactory to describe the behavior of the system, but it lacks physical insight. It is better to approach the data interpretation with a physical finite element model avoiding the nonphysical electric circuit model.

**Table 2:** Electrochemical Impedance Spectroscopy Data for Three Ionic Diode Devices^a^

element	units	diode 1	diode 2	diode 3
*R*1	KΩ	3.77 (±0.08)	3.92 (±0.07)	3.89 (±0.07)
*R*2	MΩ	1.49 (±0.01)	0.611 (±0.005)	0.900 (±0.004)
*R*3	KΩ	413 (±11)	227 (±6)	243 (±9)
*C*1	nF	0.44 (±0.002)	0.310 (±0.002)	0.420 (±0.002)
*Q*1	s^N^ Ω^–1^	6.7 (±0.3) × 10^–8^	15.7 (±0.8) × 10^–8^	32.6 (±1.6) × 10^–8^
*N*	dimensionless	0.783 (±0.008)	0.779 (±0.008)	0.700 (±0.008)

a In 10 mM NaCl; bias of 0.0 V; amplitude of 25 mV. The fitting error is given in brackets.

### 4.2 Aquivion-Coated Microhole Diodes: Computer Simulation of Steady State Phenomena

**Figure 5:**
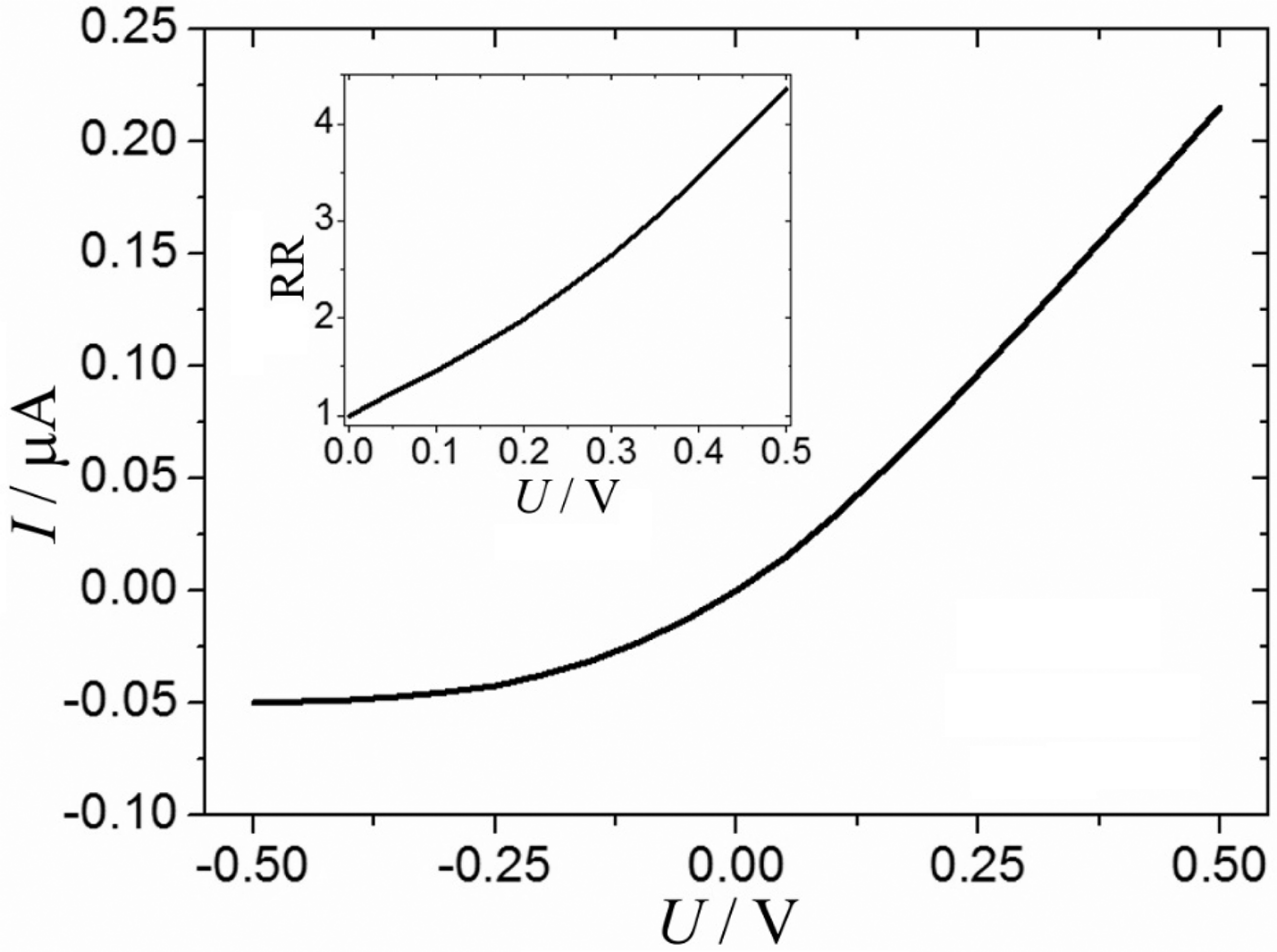
Simulated voltammetry of the ionic diode at 0.2 V s^–1^. Inset shows the rectification ratio (RR) as a function of the applied voltage.

The finite element simulation approach was based on two free parameters, *D*_Cl,mem_ and *D*_Na,mem_, which were adjusted to give a satisfactory match in the impedance data (*vide infra*). Figure 5 shows a simulated *I*–*V* curve in the range of −0.5 to 0.5 V simulated with a scan rate of 0.2 V s^–1^. The diode character and rectification effect are clearly observed (compare with Figure 4A). In the inset of Figure 5 the rectification ratio is plotted versus applied voltage (e.g., at ±0.3 V the rectification ratio is *I*(+0.3 V)/*I*(−0.3 V) = 2.5). The value increases from 1 to about 4, which is slightly lower compared to the experimental data (mainly due to the lower charge density chosen in the simulated ionomer). This parameter is important as a measure of the efficiency of the rectification performance of the diode.

### 4.3 Aquivion-Coated Microhole Diodes: Computer Simulation of Transient Phenomena

**Figure 6:**
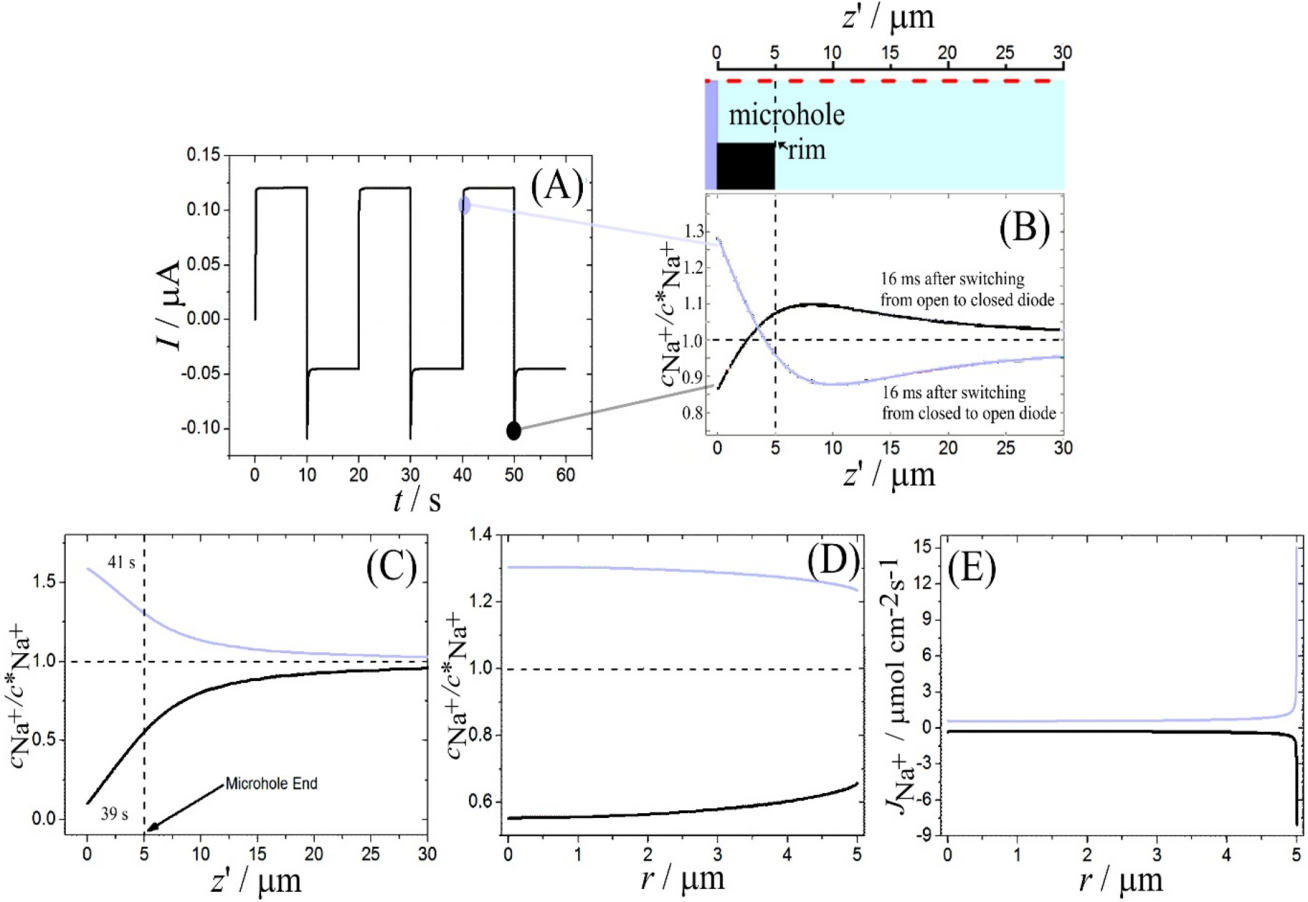
(A) Chronoamperometry data simulated for a single microhole diode at ±0.3 V. (B) During the transient response: Na^+^ concentration profile sampled at 16 ms after switching from closed to open (green) and 16 ms after switching from open to closed (black). (C) At quasi-steady state: Na^+^ concentration profile at 39 s (closed diode, black) and at 41 s (open diode, green). The dashed line indicates the rim of the microhole. (D) Plot of Na^+^ concentration (at 39 s and at 41 s) as a function of radius at the microhole rim *z*′ = 5 μm. (E) Normal flux of Na^+^ as a function of *r* at the rim of the microhole.

Figure 6A shows the simulated current that flows through the device when the applied potential is switched between −0.3 and +0.3 V. The transient currents are comparable to experimental transient data (Figure 4B, diode 2). The ratio of the current in the open state divided by the current in the closed state gives the rectification ratio (RR), which is 2.6 under these conditions. One of the advantages of the simulation is that it allows a deeper understanding of the system under investigation by access to local phenomena such as concentration profiles. Figure 6B shows a plot of the Na^+^ concentration profile (the concentration is normalized relative to the bulk concentration c*_Na+_) inside of the microhole versus the distance from the ionomer *z*′ (see Figure 1). Due to electroneutrality, Na^+^ and Cl^–^ concentrations are equal. At a time *t* = 39 s (Figure 6C; closed diode; black line), depletion occurs and the electrolyte is removed from the microhole region. In contrast, at time *t* = 41 s (Figure 6C; open diode; green line), electrolyte is accumulated inside of the microhole. Note that the parameter *z*′ represents the distance from the ionomer surface inside of the microhole. Both the gradual rise in current when switching the diode open and the negative current spike when switching the diode to closed are associated with electrolyte entering the microhole region (accumulation) and electrolyte being removed from the microhole region (depletion).

It is possible to explore the exact moment when the switching from one diode state to another occurs. Figure 6B shows the Na^+^ concentration profile 16 ms after switching from closed to open (green) and 16 ms after switching from open to closed (black). This is the moment when the net concentration of Na^+^ integrated over the microhole region is equal to the bulk electrolyte concentration. For a 10 mm diameter microhole, the diode time constant τ_diode_ = 1/ω_diode_ = 1/(2π*f*_diode_) = 16 ms can be estimated from the electrochemical impedance spectroscopy data (*vide infra*). The corresponding currents are indicated in Figure 6A as ±100 nA.

Figure 6D shows the Na^+^ concentration profile radially at the location of the rim of the microhole. The switch from depletion (39 s, red) to accumulation (41 s, green) is clearly observed. The microhole geometry produces very high fluxes at the rim of the microhole (comparable to the situation of high current densities at the edge of microelectrodes.^30^Figure 6E shows the flux of Na^+^ at the rim of the microhole and as a function of radius. For both accumulation (open diode) and depletion (closed diode), the effect of high flux at the edge is clearly observed.

### 4.4 Aquivion-Coated Microhole Diodes: Computer Simulation of Electrochemical Impedance Phenomena

Next, electrochemical impedance spectroscopy data are simulated for an applied bias of 0 V and for an amplitude of 25 mV (peak-to-peak). The first semicircular feature in the experimental data (Figure 4C) is due to charging of the Teflon film and therefore not part of the computer simulation model. The R2–C1 component of the equivalent circuit was subtracted from the experimental data to leave the second semicircular feature to be reproduced with the finite element simulation model. The resistors R1 and R2 also are not accessible in the simulation model and are therefore adjusted here with an arbitrary resistor R0.

**Figure 7:**
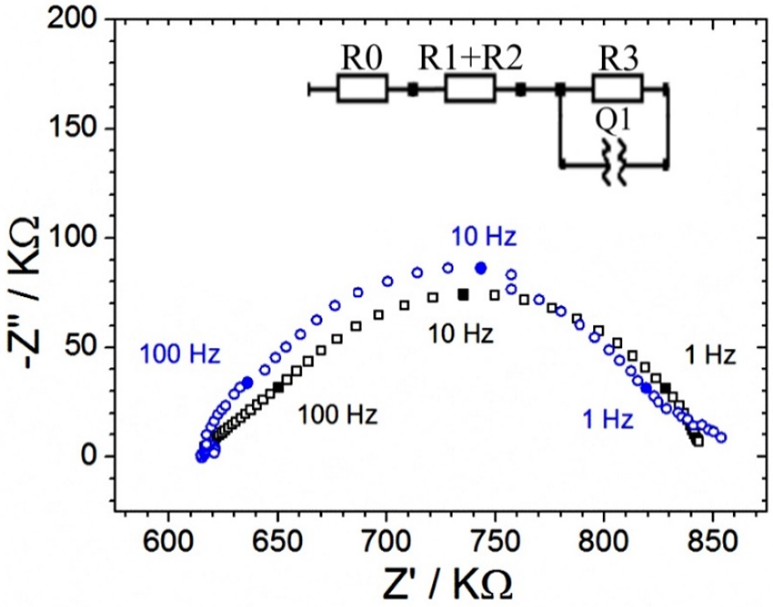
Nyquist representation of the simulated electrochemical impedance from 0.1 Hz to 10 kHz with 25 mV amplitude (black circles). Experimental data for the second “depressed” semicircular feature of diode 2 (blue circles). Inset shows the parameters fitted to the experimental data (blue) and the parameters fitted to the simulated data (black) to compared data sets (circuit shown as inset).

Table 1 summarizes all the parameters employed in the simulation with only two “free” parameters: the Na^+^ diffusion coefficient in the ionomer membrane and the Cl^–^ diffusion coefficient in the ionomer membrane. Both of these free parameters were manually adjusted to generate a satisfactory fit of the simulation data with the experimental data. Figure 7 shows a comparison of simulation and experimental data for diode 2. Values optimized for the free parameters were *D*_Na,mem_ = 6.5 × 10^–11^ m^2^ s^–1^ and *D*_Cl,mem_ = 8.4 × 10^–10^ m^2^ s^–1^. These values are physically plausible (but potentially impacted on by the too low charge density chosen for the ionomer in the simulation, *vide supra*), but inconsistent with earlier estimates for Nafion.^31^ In the low frequency limit, the simulation model is flawed due to the limited reservoir size of only 160 μm. However, most of the data fits well, confirming that the simulation does indeed reflect the conditions in the switching ionic diode. Note the “summit frequency” or diode frequency at approximately 10 Hz for both experiment and simulation. Summarized in Table 3 are equivalent circuit fitting parameters to compare both the experimental and the simulation data.

**Table 3:** Electrochemical Impedance Spectroscopy Data (in 10 mM NaCl; Bias 0.0 V; Amplitude 25 mV) Summarized in Terms of Equivalent Circuits for Ionic Diode 2 (for the Second Semi-Circular Feature) Comparing Experiment and COMSOL Fitting

circuit element	units	diode 2: experiment	diode 2: COMSOL fitting
*R*0	MΩ		–2.86
*R*1 + *R*2	ΜΩ	0.61	3.48
*R*3	KΩ	227	229
*Q*1	s^N^ Ω^–1^	15.7 × 10^–8^	22.9 × 10^–8^
*N*	dimensionless	0.779	0.707

**Figure 8:**
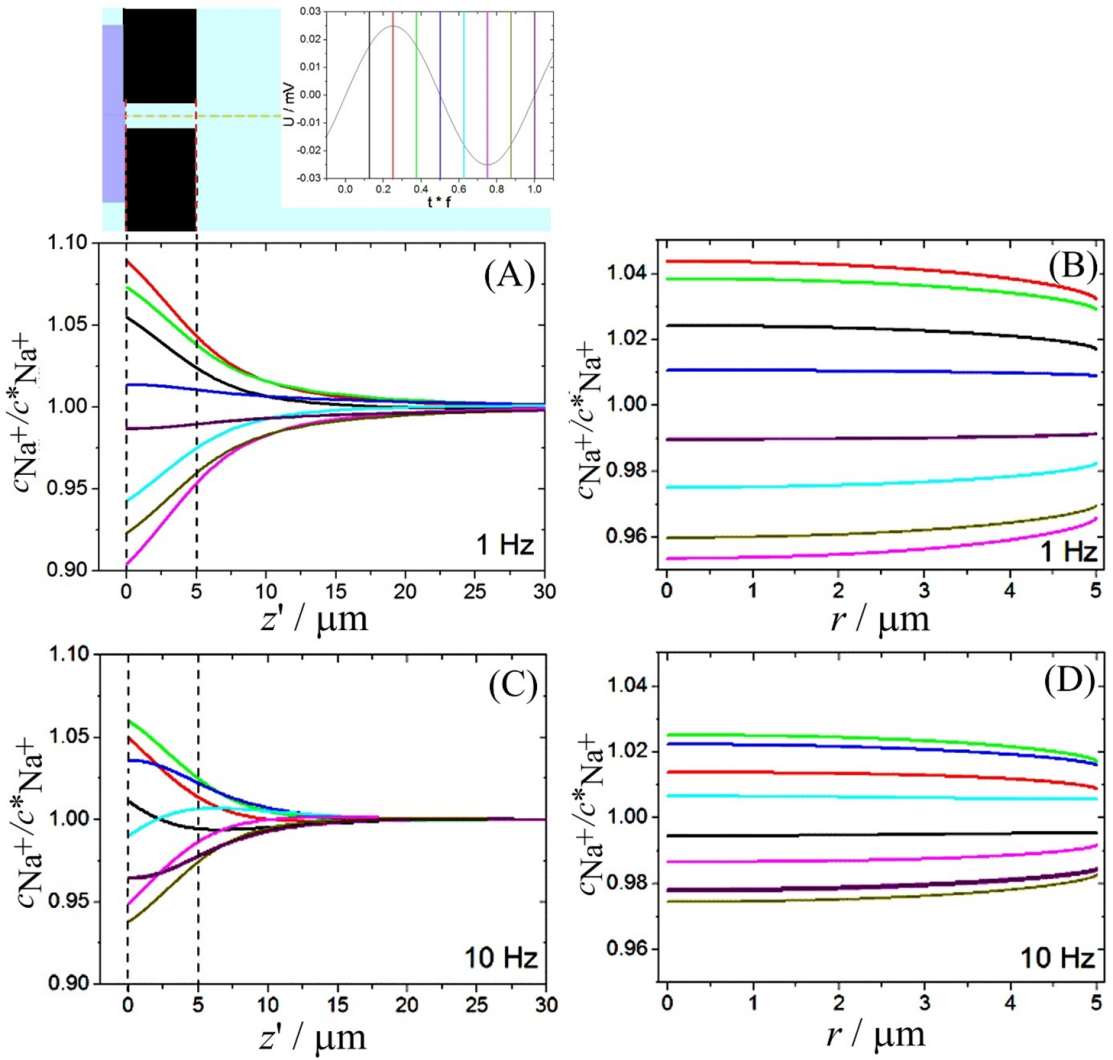
(A) Concentration profiles for Na^+^ sampled at the center of the microhole in the axial direction from the interface between the ionomer film and the electrolyte (*z*′ = 0). The dashed line indicates the rim of the microhole. (B) Na^+^ concentration profiles at the ionomer surface (*z*′ = 5 μm) at the rim of the microhole filled with electrolyte; frequency 1 Hz. (C) Concentration profiles versus *z*′ at 10 Hz. (D) Na^+^ concentration profiles versus *r*. Insert: Each curve represents a fraction of the period () related by colors  (black),  (red),  (green),  (blue),  (cyan),  (magenta),  (dark yellow), and  (purple).

Figure 8 shows the simulated sodium cation concentration profiles (normalized) over the axial and radial coordinates. Figure 8A,B show the concentration fluctuation along the *z*′ axis for a frequency of 1 Hz (see Figure 8C,D for 10 Hz data). At 1 Hz, the concentration profile is in phase with the perturbation in potential, which indicates that the diode is always close to the steady state. When the frequency is increased to 10 Hz, the changes in the concentration profile are smaller than the previous frequency, and they are no longer in phase with the perturbation in potential. In fact, at the diode frequency *f*_diode_ of the semicircular feature, at 10 Hz, a maximum phase angle is observed.

**Figure 9:**
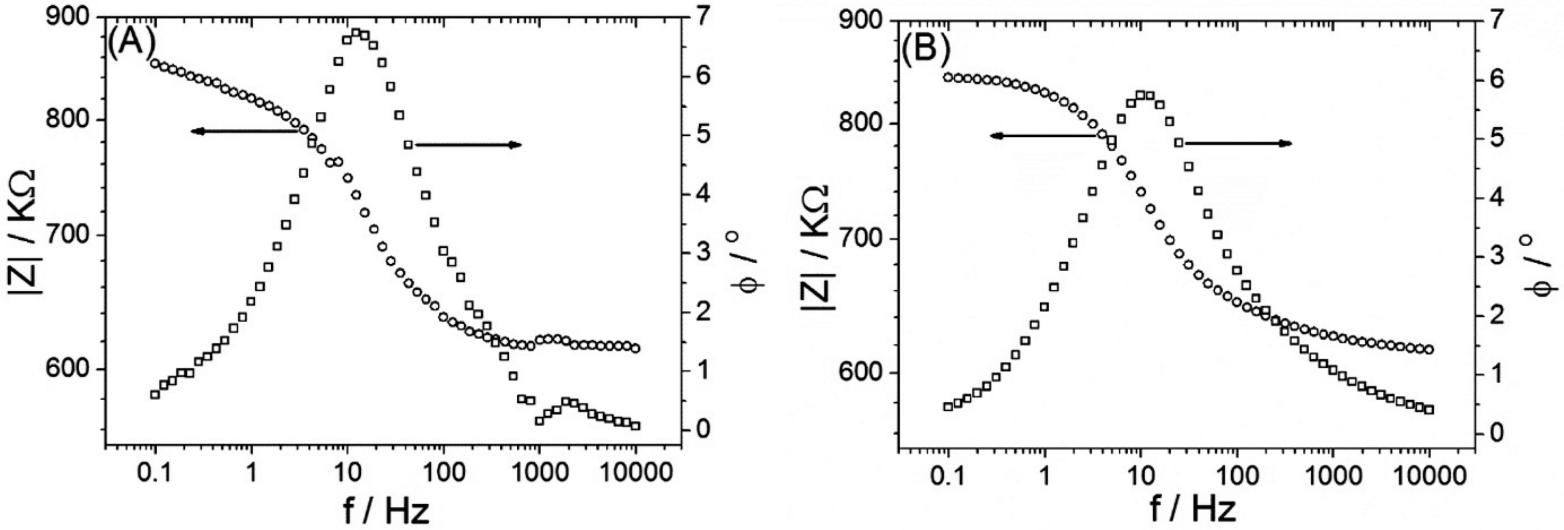
Bode plot for (A) experimental data and (B) simulation data. The summit frequency is consistent with the peak in the phase angle spectrum.

Figure 9 shows Bode plots of data from experiment (Figure 9A) and simulation (Figure 9B). The peak in the phase angle plot (consistent with the point of inflection in the |*Z*| plot) represents the point in the frequency domain for which diode switching decouples from the excitation signal (going to higher frequencies). The “summit frequency” in the Nyquist representation can therefore be understood as the frequency at which the diode switching becomes effective (going to lower frequencies).

Therefore, both the “summit frequency” *f*_diode_ in the Nyquist representation (diode frequency) and the corresponding time constant of the semicircular feature with τ_diode_ = 1/ω_diode_ = 1/(2π*f*_diode_) describe the flux of electrolyte into and out of the microhole region. The time constant τ_diode_ can be understood as the point in time where a switch between accumulation and depletion occurs, and the characteristic diode frequency *f*_diode_ represents the point in the frequency domain with maximum phase shift.

## 5 Conclusions

Transient currents and electrochemical impedance spectroscopy applied to ionic diode processes for microhole devices have been investigated with a finite element computer modeling approach. Rectification phenomena have been reproduced, and ionic diode time constant and diode frequency have been discussed in terms of physical processes and parameters. Results lead to a deeper understanding of the diode function, revealing ion concentrations as a function of time and space in the microhole during potential switching. The diode switching and the diffusion–migration of electrolyte ions are quantitatively reproduced, and the corresponding impedance spectroscopy data from experiment and from theory are in good agreement. This approach quantitatively links data to physical phenomena and goes beyond the conventional use of circuit elements to fit impedance spectroscopy data. It is now possible to explore the ionic diode performance at the level of theory to predict avenues for performance improvement.

In the future, better finite element models will be possible with higher resolution meshing to provide a better modeling of the ionomer|electrolyte interface and to better reflect the true charge density in ionomer materials and processes within the ionomer film. These models will provide theoretical tools to predict ionic diode performance and to explore performance of coupled ionic diodes, applied for example in the effective low energy desalination of seawater. These models will also allow for a better comparison of different competing ionomer materials in the context of this application.
